# Acute presentation of a benign cystadenofibroma of the fallopian tube: a case report

**DOI:** 10.1186/1752-1947-4-181

**Published:** 2010-06-17

**Authors:** Tania S de Silva, Abhijeet Patil, Roy N Lawrence

**Affiliations:** 1 Department of General Surgery, The Great Western Hospital, Marlborough Road, Swindon, UK

## Abstract

**Introduction:**

Cystadenofibromas are rare benign tumors of the fallopian tube with only 15 reported cases worldwide. They are usually asymptomatic and are found incidentally. This case is presented on account of its rarity and to the best of our knowledge, is the first reported case of cystadenofibroma of the fallopian tube discovered during an appendicectomy.

**Case presentation:**

We report a rare case of cystadenofibroma of the fallopian tube in a 19-year-old Caucasian woman who presented with sudden onset of right iliac fossa pain. A clinical diagnosis of appendicitis was made and she was taken to the operating theater for an appendicectomy. Intraoperatively, the appendix appeared normal. However, the 8 cm cyst contained within the right ovary and the blood in the pelvis warranted a salpingo-oopherectomy. Our patient made an uneventful recovery and was discharged after four days. Histology revealed a benign cystadenofibroma of the fallopian tube. There was no evidence of recurrence in the follow-up period of 12 months.

**Conclusion:**

Cystadenofibromas are benign tumors that may macroscopically and ultrasonographically appear malignant. We recommend that the diagnosis of cystadenofibroma is considered prior to performing radical surgery that may affect the fecundity of these patients. Cystadenofibromas confined to the fallopian tube can be treated curatively with unilateral salpingo-oophorectomy, without the need for any further treatment. However, long-term follow-up of more cases is required to draw more definitive conclusions.

## Introduction

Cystadenofibromas are rare benign tumors of the fallopian tube with only 15 reported cases worldwide [1]. They are usually asymptomatic and are found incidentally [2]. Sometimes they are discovered during evaluation for *in vitro *and fertilization-embryo transfer [3]. In the case of our patient, the presentation was that of an acute abdomen due to hemorrhagic necrosis of the tumor. Malignant potential is very rare. However, it may macroscopically and ultrasonographically appear malignant resulting in most of these younger women having radical surgery affecting their fecundity. It is therefore advisable to consider the possibility of cystadenofibroma prior to selecting an aggressive surgical approach in younger patients.

Here we report a rare case of a 19-year-old woman with cystadenofibroma of the fallopian tube, presenting with an acute abdomen, which was treated with a right salpingo-oophorecotmy.

## Case presentation

A 19-year-old nulliparous, British-Caucasian woman presented with a one-day history of worsening right iliac fossa pain associated with nausea and vomiting. Her previous medical and gynecological history has been uneventful. The menses of our patient (four-day duration, 28-day cycle) were regular. Abdominal examination revealed percussion tenderness over the right iliac fossa and a positive Rovsig's sign warranting an appendectomy. The white cell count and C-reactive protein levels were mildly elevated and no morphological examinations were performed. Our patient was taken to the operating theater with a view to performing an open appendectomy. However, intra-operative findings revealed free fluid in the pelvis with a normal-looking appendix. The right adenexa was found to be twisted three times and was necrotic. The ovary measured 8 cm and the fallopian tube was found to be distended and necrotic. A right salpingo-oophorectomy was performed and the left ovary was checked to be normal.

**Figure 1:**
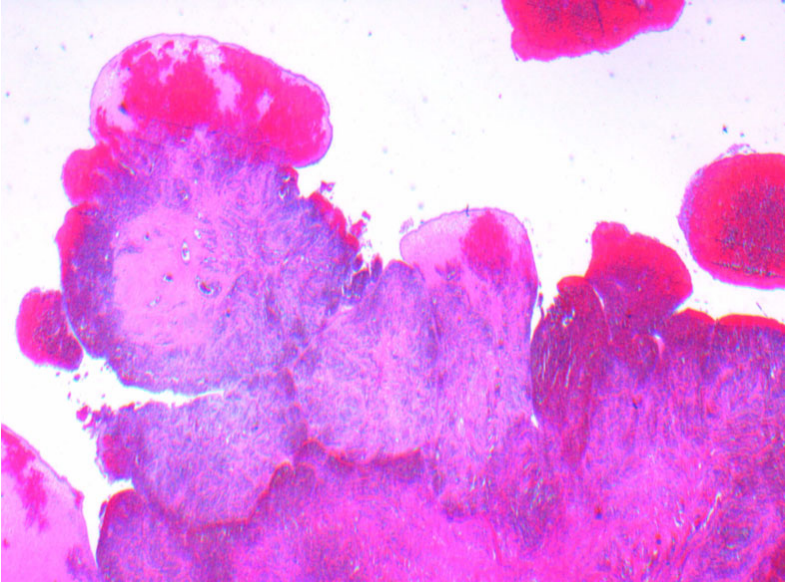
**A histology slide of right fallopian tube specimen taken at time of surgery showing congestion, hemorrhage and coagulative necrosis**.

**Figure 2:**
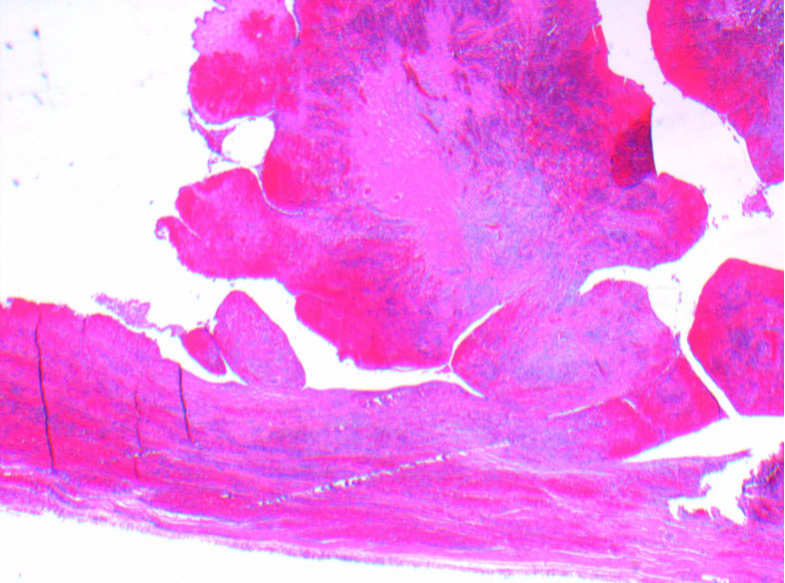
**Histology slide of right fallopian tube specimen taken at time of surgery**. The broad based papillary lesion, showed a fibrotic stroma forming broad leaf like projections lined by a low cuboidal ciliated epithelium.

**Figure 3:**
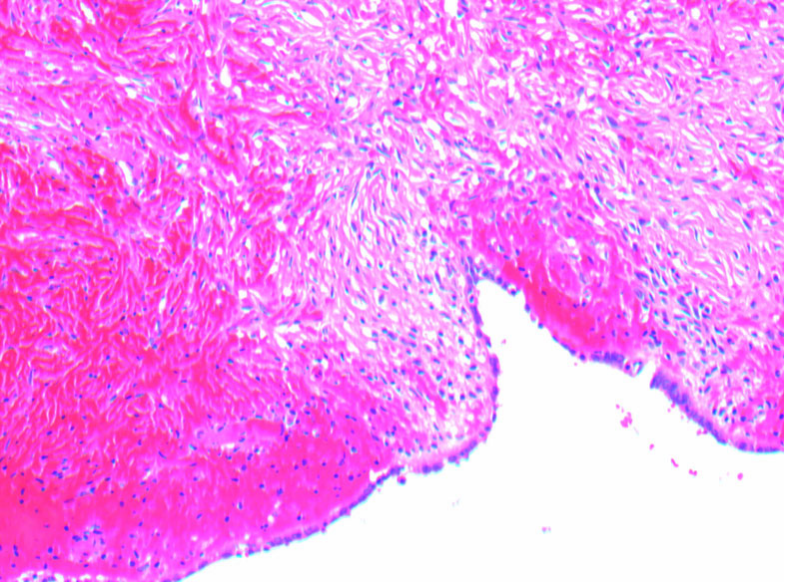
**A histology slide of right fallopian tube specimen taken at time of surgery showing fibrotic stroma lined by cuboidal epithelium**.

As seen in Figure 1, the histological analysis showed congestion, hemorrhage and coagulative necrosis of the fallopian tube. The broad-based papillary lesion showed a fibrotic stroma forming broad leaf like projections lined by a low cuboidal ciliated epithelium as seen in Figures 2 and 3. These features are consistent with benign cystadenofibroma. The ovary also showed severe hemorrhage and congestion with coagulative necrosis.

Our patient had an uneventful recovery and was discharged after four days. She has since been followed up in the out-patients clinic over a 12-month period, and was found to do well with no evidence of recurrence of disease.

## Discussion

Adenofibromas are relatively rare benign tumors with rare malignant potential, arising from the germinal lining and ovarian stroma. The relative amounts of the epithelial and stromal constituents and the secretary activity of the epithelial component will determine the solid, semisolid or liquid state of the tumor. The majority of the reported adenofibromas are of the serous type. However, endometrioid, clear cell and mucinous types also exist [4].

Having performed a Medline database search with the keywords "cystadenofibroma" and "fallopian tube", we analyzed the relevant articles and their cited references, in order to construct Table 1. Table 1 shows a systematic review of the cases of cystadenofibroma, specifically arising from the fallopian tube. During this search we encountered a number of cases of the tumor arising within the ovary, which were excluded from Table 1. The search revealed only five cases previously reported in the English literature. Clinico-pathological features of these tumors, including the current case, are summarized in Table 1.

**Table 1:** Summary of current cases and their clinico-pathological features of these tumors

	Author	year	Age	Clinical symptoms	Surgical intervention	Outcome	Pathology
[11]	Silverman AY	1978	36	Finding during tubal ligation following termination of pregnancy	Bilateral partial salpingectomy		3.5 cm cyst with small papillary projections supported by central cores of fibrous tissue and covered with ciliated cuboidal to columnar epithelial cells

[12]	Valerdiz CS *et al.*	1989	49	Incidental finding during salpingo oophorectomy for leiomyomas	Salpingo oophorectomy	WED after unspecified time	cystadenofibroma

[12]	Valerdiz CS *et al.*	1989	32	Incidental finding during early pregnancy	salpingectomy	WED after unspecified time	2.5 cm cyst with papillations- Borderline cystadenofibroma

[2]	Gurbuz Y *et al.*	2003	48	Incidental finding in woman with leiomyoma uterei	?Salpingectomy		2 serous papillary cystadennofibromas. Immunohistochemistry suggested tumor was an embryonic remnant of mullerian duct

[3]	Sills ES *et al.*	2003	Infertility, discovered during evaluation for IVF	Laparoscopic decompression and removal of intact cyst	WED after 3 months follow-up		Benign serous cystadenofibroma

	De Silva *et al.*	2009	19	Acute onset right iliac fossa pain	Open salpingo oophorectomy	WED after 12 months	Benign cystadenofibroma of fallopian tube and coagulative necrosis of ovary

IVF, *in vitro *fertilization; WED, Well enough for discharge from clinic.

Review of the current literature suggests that cystadenofibromas generally present in the fourth and fifth decades in the life of a patient. However, they appear to present earlier in a subset of women exposed to antenatal diethylsilbestrol [5].

The presenting symptoms of this tumor include abdominal pain, increased abdominal girth, dysuria, rectal urgency, vaginal bleeding and feminization [6]. One school of thought suggests that the feminization and vaginal bleeding symptoms are due to excessive estrogen secretion by the tumor causing abnormal endometrial growth [7,8]. However, other authors failed to prove excessive endometrial growth [7,9,10].

The diagnosis of cystadenofibroma is a difficult one, as they macroscopically and ultrasonographically appear malignant. They may grow up to 20 cm in diameter, encapsulated and multiloculated with short broad papillary projections. Laparosopy may also be used in the diagnosis and even treatment of this condition as demonstrated by Sills *et al*. In this paper, they described how the cyst was decompressed and removed intact without incident via a 5 mm laparoscopic cannuala [3].

Czernobilsky *et al*. studied 34 patients with benign serous cystadenofibromas and found the same favorable outcome in all patients irrespective to whether they underwent conservative cystectomy, oophorectomy or total abdominal hysterectomy and bilateral salpingo-oophorectomy [10].

## Conclusions

Cystadenofibromas of the fallopian tube are benign tumors with rare malignant potential. Therefore, we advise to consider this diagnosis before employing radical surgery in younger women, as this would impact their fertility. However, in patients over 50 years of age, presenting with any ovarian or fallopian tube tumor, there is no need for a conservative approach. In the treatment of younger patients of childbearing age, we found salpingo-oophorectomy to be curative without the need for any further treatment. However, long term follow-up of more cases are required to make more definitive conclusions.

This case is presented on account of its rarity and we believe this is the first reported case of cystadenofibroma of the fallopian tube to present acutely and discovered during an appendicectomy.

## Patient's perspective

I write the following to provide assistance to the case report written about my operation. I have no medical knowledge or background so I only write from my own perspective and experience.

Before the morning I was taken to hospital I had never experienced abdominal pains, either related to my menstrual cycle or other. I had never been submitted to hospital for any previous health concerns. It was the summer after my first year at University, I was working as a full time Assistant Director, working long hours, the job was very active and predominantly outdoors (it was an outdoor production). I was 19 years old. At the time of being submitted to hospital I was on the third day of my period, at this age I experienced regular monthly periods lasting seven days. I awoke very early on that morning with no pain. I then went back to sleep but was awoken with a severe pain in my abdomen. I also felt very hot, dizzy and clammy. I tried to recover by taking a cool bath, drinking water and then lying flat on the floor breathing deeply. This did not help and the pain began to increase to an unbearable level. An ambulance was called for, whilst waiting for them I continued to lie flat on the cool bathroom floor with the windows open.

When the ambulance arrived the ambulance woman asked if I was possibly pregnant. I said no, there was no possibility of this. They then made the presumption that it was due to drug or alcohol abuse. Again I said it was not. She then insisted it was food poisoning, I explained that the pain was far more severe than food poisoning. Finally she said that she would take me into a hospital despite not feeling it was necessary. Despite my career in theater I am not overly dramatic and despite the pain I was able to converse and I suppose did not appear to be in as much pain as I probably was. But it hurt in a way I could never put into words. I was driven to the Accident and Emergency unit. While in the ambulance I was giving a mask to breathe through and told it would help the pain; it had no affect at all. At the hospital I was put into a cubicle. A nurse then gave me an injection in my arm, I don't know what of. Whatever it was it relieved the pain instantly. I could literally feel the pain dissolve as I was given the injection - it was a heavenly experience and a great relief. A doctor then visited me and began to apply pressure to my abdomen, asking if I was in pain whilst he put pressure on different areas. I explained that when he pressed down on my abdomen, it did hurt. The pain I experienced was mainly on the right lower side. Again the doctor suggested I had food poisoning; I had gone to a barbecue the night before. I was taken up to the ward and it was then that it was suggested I possibly had appendicitis, I cannot remember much of this period up until it was decided that I be operated on. I drifted in and out of sleep and in severe pain. The morning of my operation I did not feel in as much pain as when I first entered hospital, but felt physically washed out and very tired. I remember seeing the consultant who said I looked very grey and that it was necessary to operate and remove my appendix. I was taken down to theater and awoke later. It was then explained to me that my appendix was removed, but also my right ovary and fallopian tube. I was connected to a morphine drip, which I controlled and used a lot. The next morning I was taken to have an X-ray so that they could find out what was wrong, this was until I explained that I had already had an operation. Most nights I would be sick after eating a small amount of toast and ice cream during the day. I went home after a few days, which I strongly pushed for because it was very uncomfortable being in hospital on a ward with lots of elderly ladies. I spent approximately three weeks recovering at home. After about a week a stitch in my appendix scar became infected, literally the wound bled severely and I was taken to my local hospital, where they squeezed the wound until the stitch came out. Apart from this my recovery had no problems, it was uncomfortable to sleep, and I couldn't eat strong flavored food and felt tired. I returned to University at the end of September, I took it easy and felt delicate for a further four weeks until feeling fully back to health by the end of October. I have two scars to remind me of my experience, but both healed well. I do now suffer from minor pain each month before my period begins, which I never did before the operation.

## Consent

Written informed consent was obtained from our patient for the publication of this case report and any accompanying images. A copy of the written consent is available for review by the Editor-in-Chief of this journal.

## Competing interests

The authors declare that they have no competing interests.

## Authors' contributions

Our patient was admitted under the care of RL during this episode and was followed up in outpatients' clinic. AP and TSdS were major contributors in writing the manuscript. All authors read and approved the final manuscript.
